# Culture expansion of adipose derived stromal cells. A closed automated Quantum Cell Expansion System compared with manual flask-based culture

**DOI:** 10.1186/s12967-016-1080-9

**Published:** 2016-11-16

**Authors:** Mandana Haack-Sørensen, Bjarke Follin, Morten Juhl, Sonja K. Brorsen, Rebekka H. Søndergaard, Jens Kastrup, Annette Ekblond

**Affiliations:** Cardiology Stem Cell Centre, The Heart Centre, Rigshospitalet University of Copenhagen, Juliane Maries Vej 20, Dept. 9302, 2100 Copenhagen, Denmark

**Keywords:** Adipose derived stromal cells, Cell culture, Coating, Cryoprecipitate, Mesenchymal stem cell, Storage, Bioreactor, Clinical application, Cell expansion

## Abstract

**Background:**

Adipose derived stromal cells (ASCs) are a rich and convenient source of cells for clinical regenerative therapeutic approaches. However, applications of ASCs often require cell expansion to reach the needed dose. In this study, cultivation of ASCs from stromal vascular fraction (SVF) over two passages in the automated and functionally closed Quantum Cell Expansion System (Quantum system) is compared with traditional manual cultivation.

**Methods:**

Stromal vascular fraction was isolated from abdominal fat, suspended in α-MEM supplemented with 10% Fetal Bovine Serum and seeded into either T75 flasks or a Quantum system that had been coated with cryoprecipitate. The cultivation of ASCs from SVF was performed in 3 ways: flask to flask; flask to Quantum system; and Quantum system to Quantum system. In all cases, quality controls were conducted for sterility, mycoplasmas, and endotoxins, in addition to the assessment of cell counts, viability, immunophenotype, and differentiation potential.

**Results:**

The viability of ASCs passage 0 (P0) and P1 was above 96%, regardless of cultivation in flasks or Quantum system. Expression of surface markers and differentiation potential was consistent with ISCT/IFATS standards for the ASC phenotype. Sterility, mycoplasma, and endotoxin tests were consistently negative. An average of 8.0 × 10^7^ SVF cells loaded into a Quantum system yielded 8.96 × 10^7^ ASCs P0, while 4.5 × 10^6^ SVF cells seeded per T75 flask yielded an average of 2.37 × 10^6^ ASCs—less than the number of SVF cells seeded. ASCs P1 expanded in the Quantum system demonstrated a population doubling (PD) around 2.2 regardless of whether P0 was previously cultured in flasks or Quantum, while ASCs P1 in flasks only reached a PD of 1.0. Conclusion: Manufacturing of ASCs in a Quantum system enhances ASC expansion rate and yield significantly relative to manual processing in T-flasks, while maintaining the purity and quality essential to safe and robust cell production. Notably, the use of the Quantum system entails significantly reduced working hours and thereby costs.

**Electronic supplementary material:**

The online version of this article (doi:10.1186/s12967-016-1080-9) contains supplementary material, which is available to authorized users.

## Background

Experimental stromal cell therapy is rapidly emerging and provides opportunities for the treatment of diverse degenerative disorders and has progressed into the clinical trial arena for a variety of diseases [1]. Among mesenchymal stromal cells (MSCs) that have been thoroughly investigated, adipose derived stromal cells (ASCs) have shown to be one of the most promising stromal cell types with several advantages compared to other stromal cells. ASCs can be obtained with minimally invasive procedures such as liposuction. Moreover, the primary cell populations are relatively rich in ASCs compared to, for example, MSCs from a bone marrow aspirate (BM-MSC). In vitro expansion of ASCs leads to a multipotent cell population with the capacity to differentiate into several cell lineages. ASCs secrete many different growth factors and cytokines, have immunosuppressive properties, and there are essentially no ethical concerns for their use in diverse clinical applications [2–5]. Accordingly, ASCs have been extensively explored in the last few years.

Development of effective clinical cellular therapies using human autologous or allogeneic ASCs requires manufacturing of large numbers of cells. Production of a therapeutically relevant cell product requires in vitro expansion to generate clinically relevant cell numbers. Cell processing must be performed in compliance with Good Manufacturing Practices (GMP) guidelines. This requires not only GMP-compliant reagents and materials but also standardised processes with high reproducibility [6, 7].

Conventional flask-based cell culture is commonly used for large-scale production [8–10]. However, flask-based culture expansion is time and labour intensive with many open processing steps. Owing to the adherent nature of the ASCs, expansion requires a large surface area and multiple culture flasks resulting in significant inter-flask heterogeneity and increased risk of microbial contamination [6].

Automation of cell expansion within a GMP-compliant environment may mitigate or eliminate these problems. A potential solution is the use of bioreactors, which would ideally be closed systems that integrate various cell processing and cell culture steps within a single closed device. Such bioreactor systems should expand high cell numbers with minimal manipulation. Production in bioreactors should furthermore be superior to classical flask-based cell expansion, with regard to traceability, staff workload and costs [7].

The Quantum Cell Expansion System (Terumo BCT) is a commercial GMP compliant device. The system provides automated cell culture in a functionally closed environment, which includes a hollow fibre bioreactor. Pre-defined, customizable settings dictate how cells are seeded, continuously fed, and eventually harvested.

The Quantum system has been used by other research groups for culture expansion of BM-MSCs [11–14]. In this study, we investigated for the first time culture expansion of ASCs in the Quantum system as an alternative to conventional flask-based culture for efficient clinical-scale ASC expansion.

The aim of this study was to evaluate the impact of automated and closed culture expansion in the Quantum system on ASC yield, viability, immunophenotype, purity, differentiation potential, and microbiological quality compared to traditional manual cultivation in culture flasks.

## Methods

### Experimental design

Lipoaspirates were obtained from 3 healthy female donors (age between 41 and 65 years) undergoing cosmetic surgery (Printzlau Private Hospital). Each lipoaspirate was processed to obtain stromal vascular fractions (SVF), from which ASC populations were expanded. The use of lipoaspirate has been approved by The National Committee on Health Research Ethics in Denmark, protocol no. H-3-2009-119. All donors signed an informed consent.

**Fig. 1:**
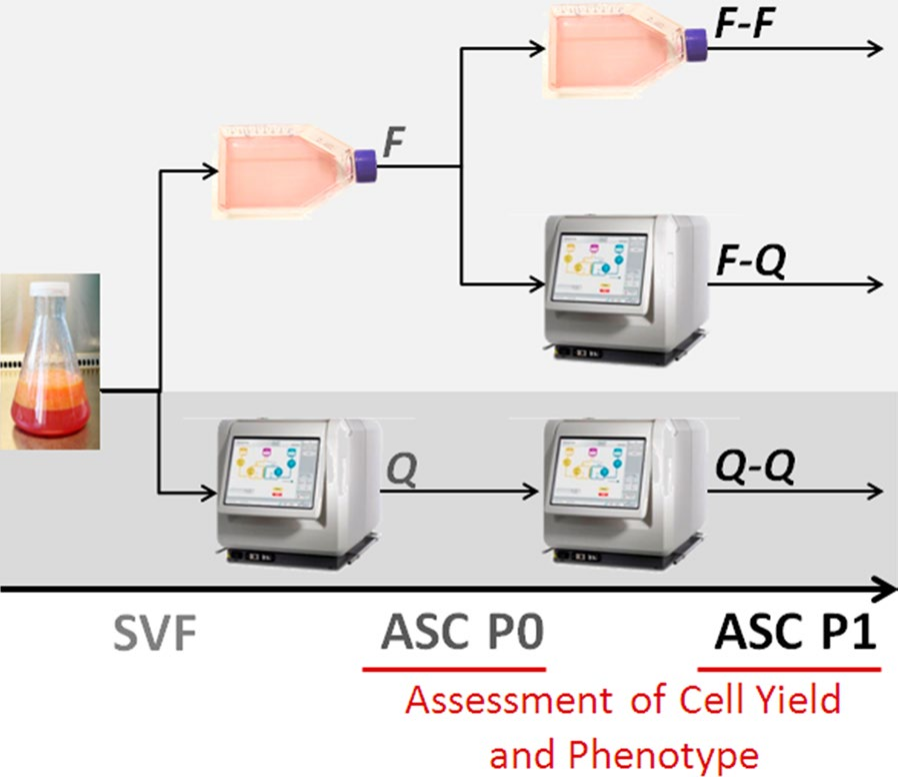
Flowchart of the experimental setup. Comparability of ASC culture expansion, P0 and P1 in flasks (*F*) and Quantum Cell Expansion System (*Q*)

The experimental setup is illustrated in Fig. 1. Expansion of ASCs was performed in a two-passage process using a traditional, flask-based process (F) or the Quantum system (Q). Primary expansion of the SVF was performed with both methods to produce ASC passage 0 (P0) cells, which were further expanded in a second passage to produce ASC P1 cells. Using the two expansion methods over two passages, three production strategies were investigated: flasks to flasks (F–F), flasks to Quantum system (F–Q), and Quantum system to Quantum system (Q–Q).

### Preparation of lipoaspirate and SVF isolation

Liposuction of subcutaneous abdominal fat was performed under local anaesthesia to provide approximately 100 ml lipoaspirate from each donor. Each lipoaspirate was washed twice with phosphate-buffered saline (PBS) (Gibco, Life Technologies) to remove residual blood. The adipose tissue was digested by incubation with 0.6 PZ U/ml collagenase NB4 (Serva GmbH) dissolved in Hank’s Balanced Salt Solution (10 × HBSS) (+CaCl_2_ +MgCl_2_, Gibco, Life Technologies) diluted to a concentration of 2 mM Ca^2+^ at 37 °C for 45 min under constant rotation. The collagenase was neutralised with complete medium containing Minimum Essential Medium, MEM Alpha (αMEM) without Ribonucleosides and Deoxyribonucleosides, (Gibco, Life Technologies), 1% Penicillin/Streptomycin (10,000 U/ml and 10.000 µg/ml, respectively) (Gibco, Life Technologies) and 10% Fetal Bovine Serum (FBS) (Gamma irradiated, Australian origin, Gibco, Life Technologies) according to a well-established protocol [15, 16] after which the suspension was filtered through a 100 µm mesh (Cell Strainer, BD Falcon), centrifuged at 1200*g* for 10 min at room temperature (RT), and re-suspended. The number of cells in the isolated SVF was counted using a NucleoCounter^®^ NC-100™ (ChemoMetec).

### Cell culture in flasks

Primary cell cultures of ASCs were established by seeding 4.5 × 10^6^ SVF cells per T75-flask (Nunc, Thermo Scientific) in complete medium. The chosen seeding density of SVF in flasks has been optimized previously in our laboratory.

The cells were incubated at standard conditions at 37 °C in humid air with 5% CO_2_. The culture medium was changed 3 days after the cells were seeded, thus removing non-adherent cells. Subsequently, the medium was changed every 3–4 days throughout the remainder of the culture period.

Reaching a confluence level of approximately 90%, the cells were harvested. For each T75 flask, the harvest procedure included an initial wash with 15 ml PBS, the addition of 3 ml TrypLE^®^ Select (Gibco, Life Technologies), incubation for 10 min at 37 °C, and neutralisation with 7 ml complete medium. The resulting suspension was centrifuged at 300*g* for 5 min at RT and re-suspended in complete medium. After counting, the cells were re-seeded at 3.5 × 10^5^ cells/T75-flask. Cell yields for ASCs at P0 and P1 were determined with a NucleoCounter^®^ NC-100™ and calculated as means of three T75 flasks.

### Cell culture in the Quantum system

The Quantum system is an automated and functionally closed system that integrates incubation, gas provision, and fluid handling for the management of a hollow fiber bioreactor. Operation of the Quantum system includes filling bags with media and reagents (e.g., media, PBS, cells, TrypLE Select), connecting these bags to the Quantum system via a sterile connection device (TSCD-II, Terumo), and controlling the system via a touch screen interface.

The Quantum system process in the current study used media and reagents that were consistent with those referenced in the “Cell Culture in Flasks” section. One additional reagent was used for coating of the bioreactor, as described in “Coating of culture surface area’’ section. Standard conditions for ASCs culture were maintained, including an incubation temperature of 37 °C and a pre-mixed gas supply (StrandMøllen) providing 5% CO_2_ and 20% O_2_, balanced with N_2_.

The Quantum system was prepared according to the manufacturer’s protocol for inserting of the disposable Cell Expansion Set (including the hollow fiber bioreactor), into the Quantum system and priming it with PBS.

#### Coating of culture surface area

Prior to loading of cells, the culture surface area of the hollow fiber bioreactor must be coated. For this purpose, 30 ml of pooled cryoprecipitate (Blood Bank, Rigshospitalet) diluted to 100 ml with PBS is loaded into the Quantum system. Coating times of 4 and 24 h were tested, in order to test if both conditions provided sufficient coating for cell attachment. Upon completion of the coating period, PBS and residual cryoprecipitate were washed out of the system and replaced with complete medium. ASCs P2 (30 × 10^6^) were loaded into the bioreactors for evaluation of the effect of coating time upon adhesion and growth.

Pools of cryoprecipitate were manufactured from fresh-frozen plasma from blood donors (blood types low titer A^+^) selected and screened according to Danish law about blood supply. Frozen plasma bags (−30 °C) were air-thawed slowly at 4 °C overnight. Plasma bags were centrifuged and cryoprecipitate separated from plasma in a CompoMat G5 blood component separator device (Fresenius KABI). The remaining cryoprecipitate was pooled from four donors and aliquoted into 30 ml portions.

#### Culture expansion of primary SVF in the bioreactor (P0)

For primary expansion of the SVF, the Quantum system was seeded with in average 80 × 10^6^ (52–100 × 10^6^) SVF suspended in 100 ml complete medium. Cells were allowed to attach for 24 h at static conditions, after which perfusion automatically began at 0.1 ml/min. After 3 days of cultivation, a washout was performed to remove any non-adherent cells.

#### Culture expansion of pre-cultured ASCs in the bioreactor (P1)

For expansion of pre-cultured ASCs (i.e., those resulting from the primary expansion), the Quantum system was seeded with 30 × 10^6^ ASCs suspended in 100 ml complete medium. A 24 h attachment period was provided, as consistent with the SVF expansion.

After the attachment period, complete medium was fed continuously into the system at increasing rates from 0.1 ml/min and gradually doubled up to 0.8 ml/min, maintaining lactate levels between 6 and 10 mM throughout the expansion. Growth of the cells was estimated based on glucose consumption and lactate production, as mentioned by Lambrechts et al. [17]. Samples from the supernatant were drawn from the sample port and glucose/lactate levels were measured with a blood gas analyzer ABL 835 FLEX (Radiometer).

#### Harvest of ASCs from bioreactor

Harvests were performed when lactate generation rates surpassed 6-9 mM/day at feeding rate 0.8 ml/min. The harvest process included a washout of the system with PBS, the addition of 180 ml TrypLE^®^ Select, and a 15 min incubation time, after which the cells were flushed into the harvest bag using complete medium. Cell yields at P0 and P1 were determined by a NucleoCounter^®^ NC-100™.

### Storage of cells

To be able to work with a single Quantum system cells need to be stored for up till 5 h while preparing a new Cell Expansion Set for successive expansion. To assess the impact of storage media on cell viability, we tested 3 different storage media, at 4 °C and RT for a 5 h period and measured viability with a NucleoCounter^®^ NC-100™ every hour. 10 × 10^6^ ASCs were re-suspended in 1 ml of: 1. Isotonic saline (9 mg/ml) (Amgros I/S) with 1% human albumin (1% HA) (20%, CSL Behring GmbH) 2. Isotonic saline with 20% human albumin (20% HA) 3. Isotonic saline with 20% autologous human serum (20% HuS).

After 5 h incubation, 1 × 10^6^ ASCs from each condition were seeded into T75 flasks. Attachment and morphology of ASCs was verified by microscopy after 24 h.

### Immunophenotyping by flow cytometry

Adipose derived stromal cells P0 and P1 culture expanded in a Quantum system or flasks were analyzed by flow cytometry (Navios, Beckman Coulter). Cells from T75 flasks were detached by incubation with TrypLE^®^ for 5–10 min at 37° C, and washed with FACS-PBS mixture [FACS-PBS (Hospital pharmacy, Copenhagen, Denmark), 1% EDTA (Hospital pharmacy, Copenhagen)], and 10% new born calf serum (GIBCO, Life Technologies). Harvested cells from a Quantum system were washed with 3 mL FACS-PBS mixture. After centrifugation (5 min at 300*g*), all cell pellets were re-suspended in FACS-PBS mixture, filtered, and distributed to FACS tubes with or without antibodies. The cells were incubated for 30 min. at room temperature with the following antibodies or isotypes: Cluster of Differentiation (CD)45-phycoerythrincyanin (PC7), CD34-allophycocyanin (APC), CD90-fluorescein isothiocyanate (FITC), CD73-phycoerythrin (PE), CD13-phycoerythrin and Texas red energy coupled dye (ECD), CD29-FITC, HLA-DR-FITC, CD19-ECD, CD14-PC7, IgG2a-FITC, IgG1-ECD, IgG1-APC, MsIgG1-PC7 (all Beckman Coulter), CD105-PE (R&D Sciences), CD166-PE, CD106-FITC, CD31-FITC, CD36-FITC, IgG1-PE, IgG1-FITC (all BD Bioscience). After incubation, the cells were washed with FACS-PBS mixture, centrifuged, and re-suspended in PBS before measuring on a Navios flow cytometer (Beckman Coulter) using a six-colour protocol. The mentioned isotype controls and Fluorescence Minus One tubes were run with each analysis. Viability was determined by addition of SYTOX blue 5 min. prior to analysis (SYTOX^®^, Invitrogen, Life Technologies). Dead cells were excluded from the final analysis, and data was analyzed using Navios software and Kaluza (Beckman Coulter).

### Differentiation potential of ASC

Adipose derived stromal cells P2 harvested from a Quantum system and flasks were tested for their ability to differentiate into osteoblasts, adipocytes and chondrocytes lineages, as described in our previous publication using StemPro differentiation kit (Gibco, Life Technology), according to the manufacturer’s protocols [18].

### Microbial contamination assays

Cell culture supernatants were collected from the Quantum system and from the culture flasks immediately before harvest. Microbial controls for bacteria and fungus were performed on supernatants using aerobic and anaerobic BacT/ALERT iFN and iFA plus culture bottles (Biomerieux) and the BacT/ALERT^®^ Microbial Detection System (Biomerieux).

Presence of mycoplasmas in cell culture supernatants were detected by PCR for mycoplasma genus at Statens Serum Institute, Copenhagen, Denmark. The content of endotoxins in end cell products were quantitatively determined by the Limulus amebocyte lysate (LAL) chromogenic method, by Statens Serum Institute.

### Statistics

Population doublings (PDs) were calculated from P1 using the following formula: PD = (log N − log N_0_)/log 2, where N is the number of harvested cells and N_0_ is the number of seeded cells.

PD calculations were shown to produce normally distributed data, when using cell counts from culture in flasks or a Quantum system. Equality of variance was confirmed by Levene’s test. A one-way ANOVA with Bonferroni correction was used for comparison of PD in Quantum–Quantum, flask–flask, and flask-Quantum in P1. All statistics were performed using IBM SPSS version 19. Graphs were made using SPSS and Excel 2010 (Microsoft Inc.). Data are depicted as mean ± Standard Error Mean in figures, and mean ± standard deviation in tables.

## Results

### Comparison of cell yield in culture flasks and Quantum system

Yield of cells obtained during P0 and P1 were compared for three donors. Loading cells in suspension into the Quantum system it is estimated that 30% of loaded volume, and thereby 30% of cells, are lost in residual space in tubes. This loss is based on a direct calculation of lines volumes according to the manufacturer; therefore only 70% of loaded cells enter the actual bioreactor for attachment and cell culture. This estimated cell loss has been taken into account in all cell yield calculations in presented results.

The cell growth in Quantum was estimated by monitoring glucose and lactate metabolism in culture media.

#### Cell yield in P0

An average seeding density of 5.56 × 10^7^ (±1.76 × 10^7^) SVF cells (corresponding to 70% of loaded cells) loaded into a Quantum system yielded 8.96 × 10^7^ (±4.88 × 10^7^) ASCs P0, which equals 1.61 times the total number of SVF cells seeded, within a 24 day culture period (mean 15 ± 8.54 days) (Table 1).

**Table 1:** Cell yields. Number of cells seeded and final number of cells obtained after ASC expansion P0 and P1 in flasks and Quantum System

n = 3	SVF seeded	SVF seeded per cm^2^	Mean days in culture	ASC P0 harvested	Viability	ASC:SVF ratio
Flask (T75)	4.50 × 10^6^	6.00 × 10^4^	8 ± 1.41	2.37 ± 0.88 × 10^6^	97%	0.53 ± 0.01
Quantum	5.56 ± 1.76 × 10^7^	2.65 × 10^3^	15 ± 8.54	8.93 ± 4.88 × 10^7^	96%	1.61 ± 0.01

n = 3	ASC P0	ASC seeded per cm^**2**^	Mean days in culture	ASC P1 harvested	Viability	PD
F–F	3.50 × 10^5^	4.67 × 10^3^	17 ± 5.57	7.53 ± 0.77 × 10^5^	98%	1.10 ± 0.15
Q–Q	2.10 × 10^7^	1.00 × 10^3^	18 ± 5.77	9.53 ± 0.58 × 10^7^	96%	2.18 ± 0.09
F–Q	2.10 × 10^7^	1.00 × 10^3^	17 ± 6.03	9.91 ± 1.28 × 10^7^	97%	2.23 ± 0.19

Results are expressed as the mean number (±SD) obtained from three donors

In T75 flasks 4.5 × 10^6^ SVF cells were seeded yielding an average of 2.37 × 10^6^ (±8.83 × 10^5^) ASCs P0, which only equals 0.53 times the total number of SVF initially seeded, within a 10 day culture period (mean 8 ± 1.41 days) (Table 1). As the exact number of ASCs contained in a heterogeneous SVF is unknown, calculation of PDs is not an option for initial passage. Of notice, to maintain a viable and proliferative culture in flasks, the seeding density of SVF cells per cm^2^ was 22 times higher in flasks compared to the Quantum system.

#### Population doublings in passage 1

The ASCs P1 in Quantum systems proliferated faster and had significantly higher PDs compared to flasks, regardless of flask or Quantum system origin (*p* < 0.05). The number of PDs for ASCs P1 passaged from Quantum to Quantum (Q–Q) was 2.18, and number of PDs for ASCs P1 passaged from flasks to Quantum (F-Q) was 2.23. ASCs P1 passaged on from flask into new flasks (F–F), reached 1.10 PDs, with a comparable number of days in culture. As for P0 cultures, seeding density of ASCs P1 was higher (approximately 5 times) in flasks than in Quantum in order to maintain viable and proliferative cultures. Viability of ASCs P0 and P1, regardless of cultivation in flasks or Quantum, was above 96% (Table 1).

### Microbial tests

Microbial tests for aerobic and anaerobic bacteria and fungus and mycoplasmas from culture supernatants were all negative, and the endotoxin concentration in all final cell preparations was less than 10 IU/ml.

### Comparison of ASC phenotype in culture flasks and Quantum system

Adipose derived stromal cell immunophenotype was documented according to the Joint statement of the International Federation for Adipose Therapeutics and Science (IFATS) and the International Society for Cellular Therapy (ISCT) [19]. Immunophenotype was identified for ASCs P0 and P1 culture expanded in flasks and Quantum systems.

#### Immunophenotype P0

**Fig. 2:**
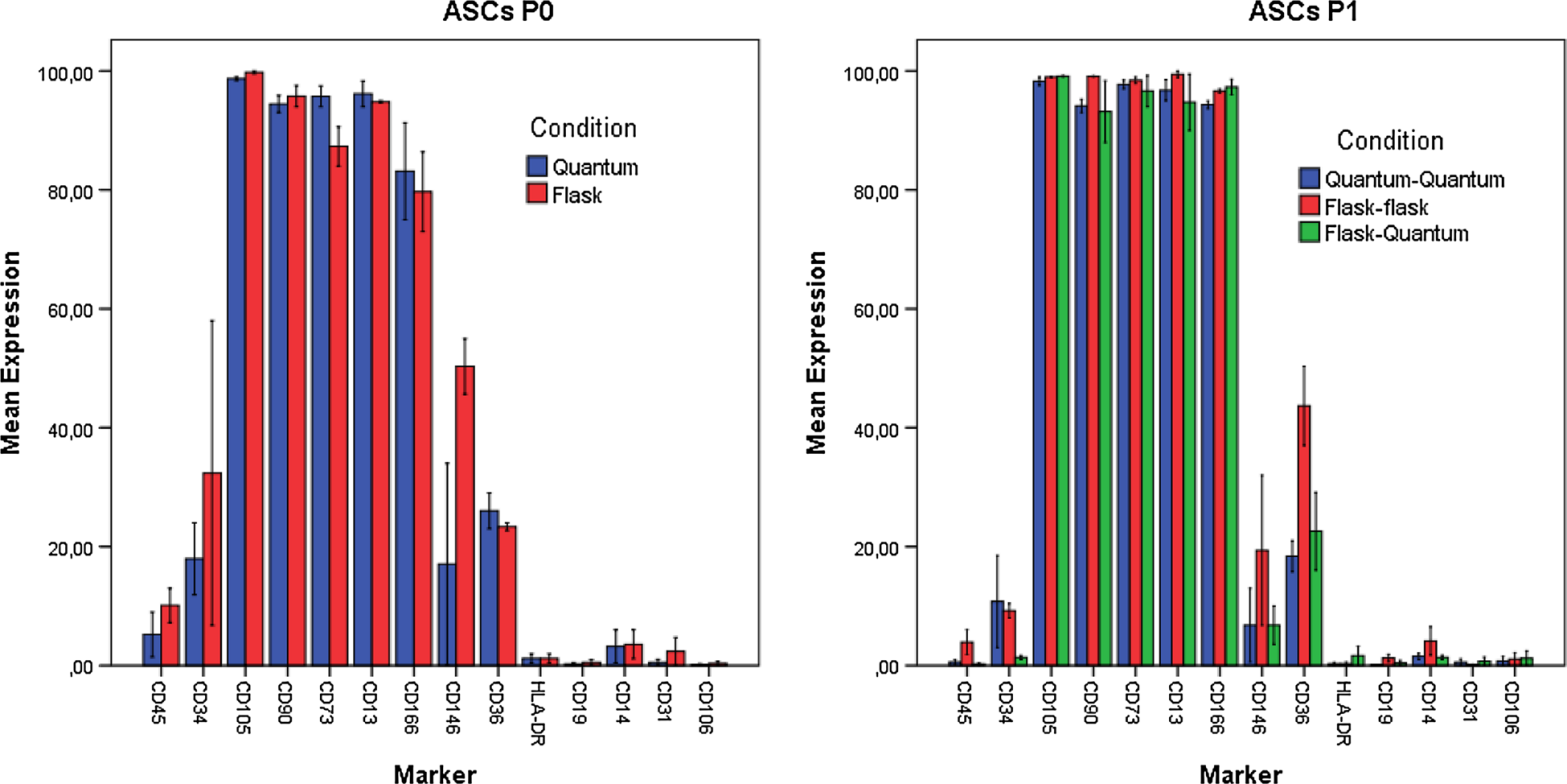
Flow cytometry immunophenotyping of culture-expanded ASCs P0 and P1 in flasks and Quantum System (n = 2). The expression is shown as percentage positive cells. Results are expressed as (mean ± SEM)

The populations of ASCs P0 from flasks and Quantum systems were almost identical. More than 80% of the cells in P0 were positive for CD105, CD90, CD73, and CD13, and around 70% for CD166. Approximately 20–30% of the P0 population was positive for CD34, CD146, and CD36, with CD146 higher levels on ASCs from flasks. CD45 and CD14 were low expressed, while HLA-DR, CD19, CD31, and CD106 were negative (Fig. 2).

#### Immunophenotype P1

After the first passage, ASCs P1 had increased expression of CD105, CD90, CD73, CD13, and CD166 to >95%, and CD45 and HLA-DR were negatively expressed for all culture conditions and was consistent with ISCT/IFATS criteria. CD34 and CD146 expressed to low levels (<20%). CD36 expression remained the same in Quantum, while it increased to approximately 40% in flasks (Fig. 2).

### Trilineage differentiation potential of ASC

ASCs P2 that was either expanded in the Quantum system or in flasks were tested for their ability to differentiate toward osteogenic, adipogenic and chondrogenic lineages. The ASC were able to differentiate into each of the three lineages regardless of previous culture conditions (Additional file 1: Fig. S1).

### Impact of Quantum system coating time on ASC yield and phenotype

**Fig. 3:**
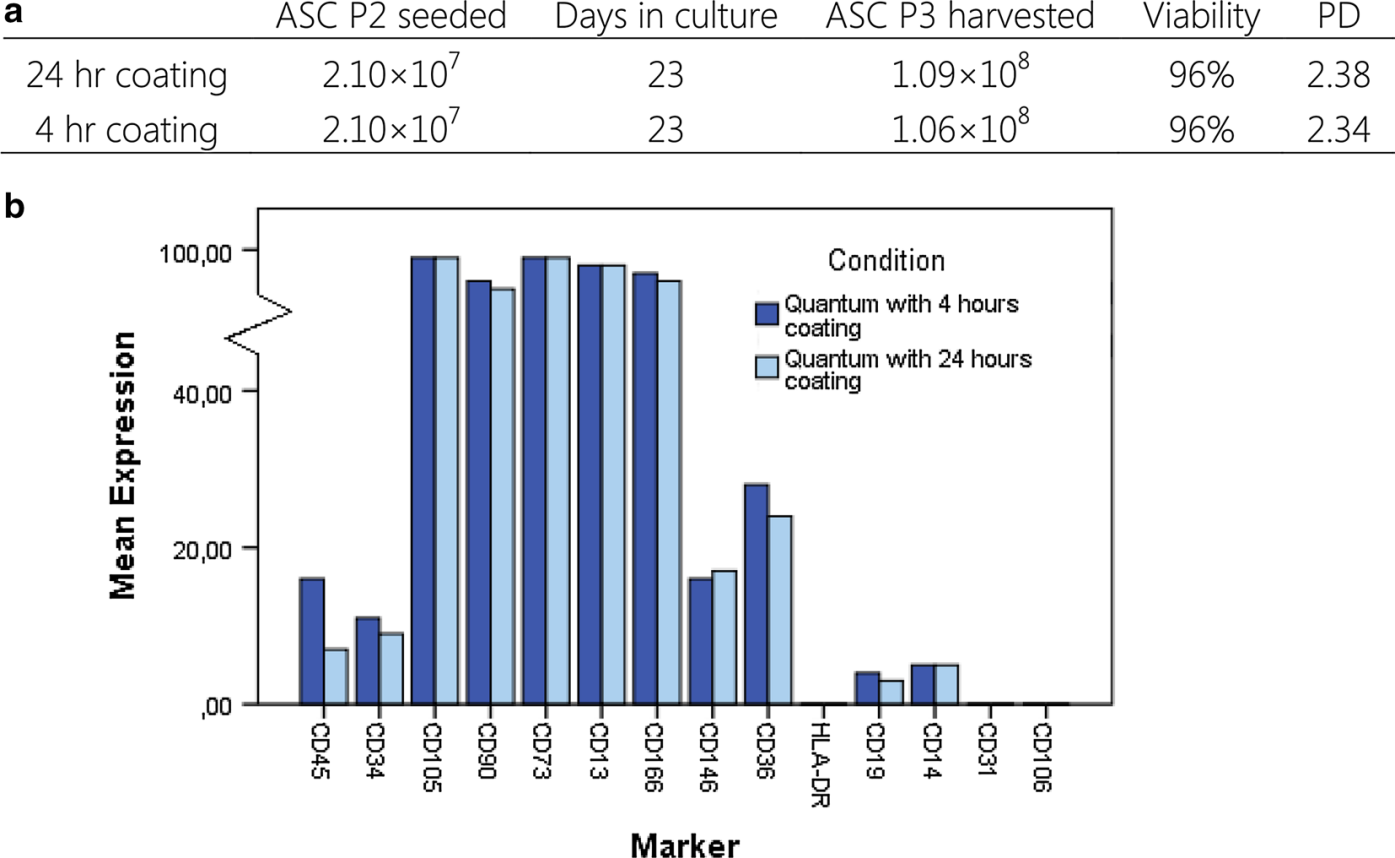
Impact of the coating time. Two bioreactors were coated with cryoprecipitate in 4 and 24 h, respectively. **a** After 3 weeks cultivation, the same amount of cells was harvested from both Quantums with similar viability and PD. **b** ASC phenotype assessed by flow cytometry on ASCs seeded in Quantum coated for 24 h compared to ASCs seeded in Quantum coated for 4 h. The expression is shown as percentage positive cells

In order to facilitate adhesion of ASCs to the hollow fibers inside the Quantum system, the fibers were coated with cryoprecipitate. To perform serial passages of fresh unpreserved cells with a single Quantum system only, the time spent on preparation for next passage needs to be short. As such the impact of a 4 and 24 h coating protocol was determined based on ASC yield and phenotype after P3 expansion. The duration of coating prior to loading of ASCs into Quantum systems did not affect cell yield. 30 × 10^6^ ASCs P2 from the same donor were loaded into two Quantum system (with 21 × 10^6^ cells reaching the hollow fibers, corresponding to 70%). After 3 weeks of expansion, 106 × 10^6^ ASCs (4 h) and 109 × 10^6^ ASCs (24 h) were harvested, respectively. Achieved PDs were 2.3 and viability was 96%, regardless of duration of coating (Fig. 3a). In addition, there was no difference in ASC phenotype depending on time spent on coating (Fig. 3b).

### Impact of pre-load storage on ASC viability

Serial passaging in a Quantum system interrupted by 4 h coating protocols require optimization of ASC storage conditions to preserve ASC viability and function while next passage is prepared.

**Fig. 4:**
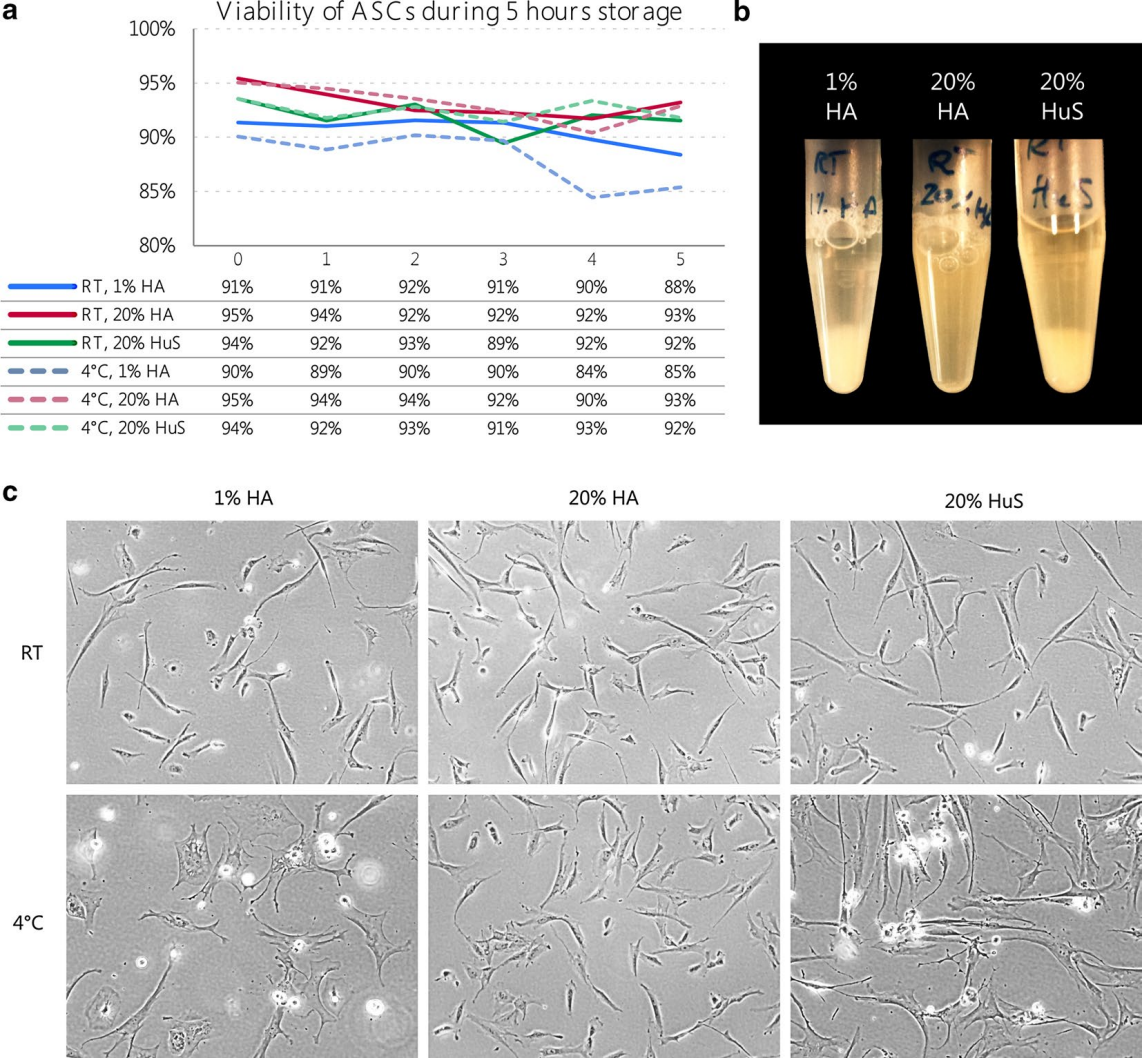
Storage of ASCs. **a** Cell viability was measured every hour, while ASCs were stored for 5 h in 3 different suspensions: 1% HA, 20% HA or 20% HuS at either RT or 4 °C. **b** Visual observation of cells stored in 3 mentioned storage media at RT. **c** ASCs from all storage conditions were seeded in culture flasks. Morphology and cell attachment ability of ASCs was evaluated after 24 h under a microscope. Representative phase contrast images at ×10 magnification. *HA* human albumin, *HuS* human serum, *RT* room temperature

The results were consistent between tested donors and showed that two of the storage media tested; 20% human albumin (20% HA) and Isotonic saline with 20% autologous human serum (20% HuS) both proved to maintain more than 90% viable ASCs after 5 h storage at 4° C as well as at RT. Storage media holding isotonic saline with 1% human albumin (1% HA) failed to maintain ASC viability above 90% during 5 h storage (Fig. 4a).

Moreover, a visual inspection of the cell suspensions after 5 h storage showed that when re-suspended in 20% HA the ASCs did not clump or sediment but were kept in suspension thereby favouring uniform re-seeding of cells (Fig. 4b).

The function of the stored ASCs was verified by adhesion tests, where 1 × 10^6^ ASCs were seeded in T75 flasks after 5 h storage and attachment of ASCs was observed under a microscope after 24 h. Morphology of the cells is shown in Fig. 4c. Most of the seeded ASCs that were stored in 20% HA and 20% HuS attached to the surface of the flasks and had a spindle-shaped and fibroblast-like morphology; however, more dead cells were visible in those flasks with 20% HuS. The adhesion capacity of re-seeded ASCs stored in 1% HA was reduced and more floating dead cells was observed compared to the other two conditions at both 4 °C and RT.

## Discussion

The success of clinical cell therapy depends on efficient, feasible, standardised, robust and reproducible manufacturing of the cell product. Currently, the gold standard for production of ASCs is flask-based. In this study, we present for the first time a comparison of ASCs culture expanded in conventional cell culture flasks and a closed Quantum system.

Adipose derived stromal cells were culture expanded over two passages in either cell culture flasks or a Quantum system. Seeding density of SVF and ASCs for expansion in flasks, which has been optimized in our laboratory, is 6.0 × 10^4^ SVF and 4.7 × 10^3^ASCs per cm^2^ in T75 flasks. In the Quantum system, according to advice from the manufacturer, a 22 times lower SVF cell density was used (2.7 × 10^3^ per cm^2^) and almost 5 times less ASC P0 (1.0 × 10^3^ per cm^2^). We do not use ASCs exceeding P1 for clinical experiments, which is why we did not test ASCs for more passages.

The fact that the total yield of ASCs from SVF is greater in Quantum systems than in flasks is not surprising, as the growth area is 280 times larger, and contact inhibition therefore is not an issue. The relevant measure for comparison of proliferation is therefore not cell yield, but PD.

During primary passage, expanding ASCs from initially seeded SVF, the exact number of ASCs seeded is not known. The SVF is a heterogeneous population of cells with an estimated content of ASCs around 2% [20]. However, calculating the ratio of ASCs obtained per SVF cell seeded, expansion in Quantum systems proves to be superior to flasks. With subsequent expansion of ASCs, the numbers of harvested ASCs P1 from Quantum systems were similar regardless of initial expansion in flasks or Quantum systems. The PDs achieved in Quantum systems were twice as high as in flasks. Studies have compared culture expansion of BM-MSC in flasks vs Quantum systems, and have reported twice as high a BM-MSC yield after two passages compared to flask cultivation [13, 14]. However, two other studies showed no significant differences in PDs when comparing culture expansion of BM-MSC in flasks and Quantum systems [12, 21]. Though the ASCs perform more extensive proliferation, they would not be suspected to be senescent. ASCs in T75 flasks with FBS medium have been expanded for more than 10 cumulative PDs after P1, without displaying signs of senescence [22].

The Quantum system clearly favours cell proliferation and yield from very small initial SVF seeding densities. This is likely explained by the dynamic culture conditions which provide continuous feeding and disposal of waste thereby reducing abrupt fluctuations in pH and local paracrine environments mediated by manual batch change of medium.

Another potential explanation for the lower PD in flasks is contact inhibition. With a limited amount of space, as in the flasks, the cells will stop proliferate rather early, resulting in a lower PD over the set time. This would occur in some sections of the flasks, even though the cells were passaged at a 90% overall confluence level [23]. To achieve similar culture conditions for ASCs in Quantum systems and flasks, the seeding density in the flasks was changed in one experiment to match the density in the Quantum system. However, the cells did not proliferate, and the ASC population never became homogeneous (data not shown).

A third potentially decisive factor is the use of cryoprecipitate in Quantum systems. Cryoprecipitate facilitates adhesion of ASCs in the bioreactor which does not have a cell culture treated hydrophilic surface like traditional T flasks. This could be the reason for the expansion of ASCs seeded in lower densities, though this is only speculations since cryoprecipitate coating has not been isolated as a factor and compared with non-coating.

All in all, since superior cell yields can be obtained using lower seeding densities, the total cell yield per donor will ultimately be greater in a Quantum system. Results also demonstrated that it is possible to start the primary passage with SVF cells in culture flasks and culture expand the subsequent passages in quantum and gain same cell yield as if SVF were cultured in Quantum system. This would be useful if the start material is too small to load a bioreactor.

Translation from manual cell culture to expansion of cells in Quantum systems demands biological coating in order to allow attachment of cells. Several studies that have used Quantum systems for culture expansion of BM-MSCs, have used fibronectin as a coating material [11–14, 21]. However, clinical grade fibronectin can be very costly. In our study, we used pooled cryoprecipitate manufactured in the Blood Bank at our hospital (Rigshospitalet, University Hospital Copenhagen). Cryoprecipitate is a concentrated blood product made from fresh frozen plasma and is in clinical use for patients with significant hypofibrinogenemia [24]. Cryoprecipitate contains diverse proteins involved in blood clotting,—fibrinogen, factor VIII, factor XIII, von Willebrand Factor and also fibronectin [24]. Viral safety and potential risk of transmitting animal spongiform encephalopathy agents is handled by donor screening and serology, and traceability of blood donors as secured through compliance with Danish law. Overall the risk of transmitting prions is negligible compared to commonly accepted use of FBS during cell culture [6].

Our results demonstrated that coating the bioreactor with cryoprecipitate for 4 h, which simplifies the setup considerably, is as effective as overnight coating with regard to cell yield and phenotype. Storage conditions however have an effect on cell survival and likely also seeding efficiency. The optimal way to store the cells while coating a new bioreactor was in 20% HA at RT, where cells did not clump or sediment and maintained normal morphology and the ability to adhere. Furthermore storage in 20% HA seemed to wash out the importance of temperature.

Several studies have addressed the short time storage of BM-MSCs in different media for immediate administration in cell therapy, such as saline or dextrose [25] or 1% HA [26, 27]. The only study which used HA in concentrations above 1%, found no difference between storage of MSCs in 1% HA and 5% HA, but observed a differences between HA from different vendors, which is potentially important in a clinical setup [27]. Over all, other studies have found good short-term viability of various MSCs regardless of used storage medium [28, 29]. However, we suggest that the ability to also maintain cells in single cell suspension is important for subsequent seeding and culture in the Quantum system.

Several critical quality attributes have been proposed for cell-based products, which include identity of the product and its potency and safety [30]. Culture expanded ASCs, regardless of culture conditions showed to be a homogeneous population with a phenotype that is in consistence with ISCT/IFATS criteria [19].

Measurements of potency remain one of the biggest challenges for cell therapy product development [31]. One of the functional criteria for the identification of ASCs drawn by ISCT and IFATS is that ASCs are capable of trilineage differentiates toward osteogenic, adipogenic and chondrogenic lineages [19, 32]. Culture expanded ASCs from flasks and bioreactor all proved trilineage potential (data not shown). This may not be the most relevant potency assay for an ASC-based therapy, as it rarely addresses the expected clinical mode of action, but additional validated and standardized potency assays are simply not yet developed. At this point we argue that viability, adhesion and proliferative capacity are the best predictors of maintained cell function and thereby clinical efficiency. These measures are contained in the present study.

One of the major risks related to the production of ASCs as well as any other cell product intended for clinical use is microbial contamination. Proper quality management and aseptic manufacturing is the focal point. Introducing the use of closed Quantum systems with automated feeding minimises the number of open manual steps considerably with a shared impact on feasibility, reproducibility and safety. Notably, open processes require the highest possible clean area classification of facilities (Class 100/ISO 5/Grade A) whereas the use of closed systems may reach approval in lower classified areas. Used clean area classification has a major impact upon feasibility and costs for set up and maintenance.

## Conclusion

Manufacturing of ASCs in a Quantum system enhances ASC expansion rate and yield significantly relative to manual processing in T-flasks, while maintaining the purity and quality essential to safe and robust cell production. In addition, the use of cryoprecipitate for coating, the short 4 h coating time, and an optimized protocol for storage of the cell during this process validates the use of a single Quantum unit for expansion of a cell product for several passages. Notably, the use of the Quantum system also provides significant reduced staff and cost.

## Additional file

**Additional file 1: Fig S1.** Differentiation of ASCs after culture in either T75 flasks or quantum system towards adipogenic, osteogenic, and chondrogenic lineage. Differentiation was evaluated by cytochemically staining with Oil Red O for adipogenic, Alizarin Red S for osteogenic, and Alcian Blue for chondrogenic.

## Abbreviations

ANOVA: ANalysis Of VAriance
ASC: adipose-derived stromal cells
BM-MSC: bone marrow MSC
CD: cluster of differentiation
FBS: fetal bovine serum
GMP: good manufacturing practice
HA: human albumin
HuS: human serum
ISCT: international society for cellular therapy
IFATS: international federation for adipose therapeutics and science
MSC: mesenchymal stromal cells
PBS: phosphate-buffered saline
PD: population doublings
MSC: mesenchymal stem/stromal cells
SVF: stromal vascular fraction
