# Supplementary material for: Culture expansion of adipose derived stromal cells. A closed automated Quantum Cell Expansion System compared with manual flask-based culture
Source: J Transl Med. 2016 Nov 16;14:319. doi: 10.1186/s12967-016-1080-9 (PMC5112664; doi:10.1186/s12967-016-1080-9)

**Supplementary Figure 1**

Differentiation of ASCs after culture in either T75 flasks or quantum system towards adipogenic, osteogenic, and chondrogenic lineage. Differentiation was evaluated by cytochemically staining with Oil Red O for adipogenic, Alizarin Red S for osteogenic, and Alcian Blue for chondrogenic.


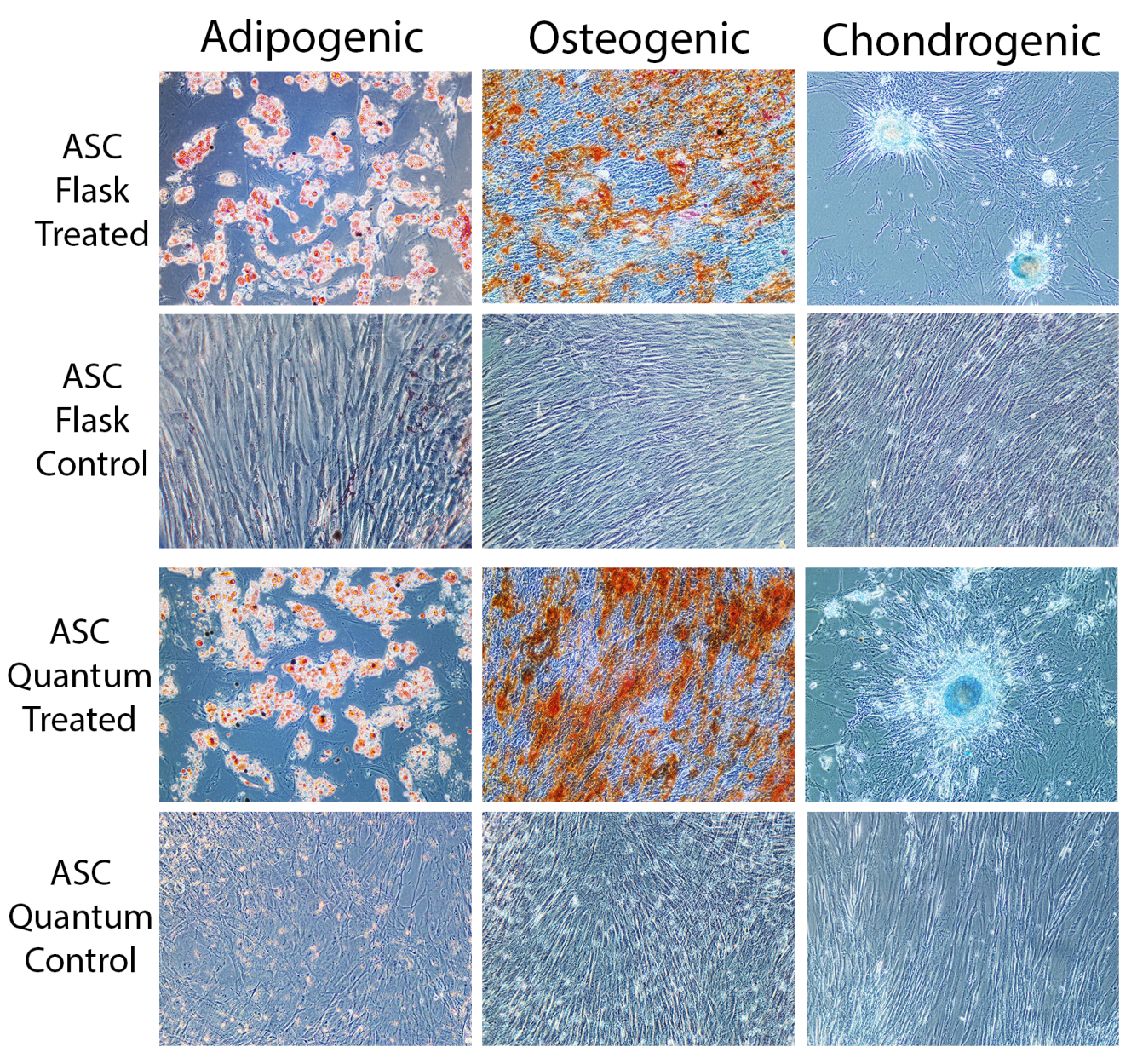

Supplement: Supplementary file 1 — Additional file 1: Fig S1. Differentiation of ASCs after culture in either T75 flasks or quantum system towards adipogenic, osteogenic, and chondrogenic lineage. Differentiation was evaluated by cytochemically staining with Oil Red O for adipogenic, Alizarin Red S for osteogenic, and Alcian Blue for chondrogenic. [file 12967_2016_1080_MOESM1_ESM.docx]
